# Disease-associated *Streptococcus suis* (DASS) in lactation: detection patterns and implications for control

**DOI:** 10.1186/s40813-025-00469-y

**Published:** 2025-11-07

**Authors:** Robert Mugabi, Ana Paula S. Poeta Silva, Cara Haden, Jerry Wells, Gemma G. R. Murray, Alex Gussak, Marisa L. Rotolo, Todd Williams, Marcelo Gottschalk, Cameron Schmitt, Maria Laura Ferrando, Peter van Baarlen, Justin Brown, Lucy Weinert, Christopher Rademacher, Ganwu Li, Rebecca Robbins, Jean Paul Cano, Locke Karriker, Perry Harms, Alexander W. Tucker, Maria J. Clavijo

**Affiliations:** 1 Veterinary Diagnostic and Population Animal Medicine, Iowa State University, Ames, IA USA; 2 Pipestone Veterinary Services, Pipestone, MN USA; 3 Host-Microbe Interactomics Group, Animal Sciences Department, Wageningen University, Wageningen, Netherlands; 4 Department of Genetics, Evolution and Environment, University College London, London, WC1E 6BT UK; 5 National Pork Board, Des Moines, IA USA; 6 Groupe de Recherche sur les Maladies Infectieuses du Porc, Faculté de médecine vétérinaire, Université de Montréal, Montréal, Canada; 7 Department of Veterinary Medicine, University of Cambridge, Cambridge, CB3 0ES UK; 8 PIC®, Hendersonville, TN USA; 9 Servicios Integrales Veterinarios, Sant Cugat del Valles, Barcelona, Spain

**Keywords:** Streptococcus suis, Disease-associated *Streptococcus suis*, qPCR, Parity effect, Swine, Medicated early weaning, Colonization dynamics

## Abstract

**Background:**

Disease-associated *Streptococcus suis* (DASS) refers to strains of *S. suis* that cause systemic infections in swine, including meningitis, septicemia, and pneumonia, resulting in significant economic losses and welfare concerns. Effective control of DASS on swine farms requires accurately detecting these pathogenic strains and identifying carrier animals and reservoirs. In this study, the dynamics of DASS colonization in clinically healthy dams, their piglets, and the surrounding environment was investigated on two commercial swine farms using novel qPCR assays: one targeting the *recN* gene (species-specific) and another targeting the *SSU_RS01130* gene (associated with disease-causing strains). The objectives were to identify optimal sampling sites, assess the impact of dam parity, and understand the disease dynamics of DASS to provide a baseline for future studies to improve control strategies.

**Results:**

Gilts and their piglets consistently exhibited higher DASS colonization compared to sows, underscoring the need for parity-based interventions. Tonsil and nasal samples were the most reliable for DASS detection in dams, while udder, fecal, and the environmental sites may serve as potential reservoirs for piglet colonization. All piglets were colonized with *S. suis* within 24 h of birth, but not all carried DASS; notably, a substantial proportion, particularly on Farm 1, remained DASS-negative at weaning. DASS detection in piglets decreased at day 7 and rebounded by day 21, reflecting dynamic colonization patterns. Farm-specific differences highlighted the impact of management practices and strain variation, with Farm 2 showing consistently higher DASS prevalence and persistence. Notably, the consistent absence of DASS in some litters suggests that targeted management, good hygiene, and dam-related factors such as parity can effectively reduce transmission risk.

**Conclusion:**

This study highlights how parity, sampling site selection, and environmental reservoirs may influence DASS colonization and persistence. This study generated valuable data that can inform future investigations aimed at improving DASS control strategies, including parity segregation, batch farrowing, maternal immunity enhancement, improved colostrum management, and hygiene protocols. Additionally, the findings support the potential refinement of Medicated Early Weaning (MEW) strategies, integrating antimicrobial use, hygiene improvements, and dam-focused interventions to reduce DASS prevalence. The novel qPCR assays offer a reliable, culture-independent surveillance tool for DASS detection, enabling veterinarians to develop evidence-based programs for early detection of, and to mitigate the impact of DASS in swine herds.

**Supplementary Information:**

The online version contains supplementary material available at 10.1186/s40813-025-00469-y.

## Background

*Streptococcus suis* is a Gram-positive coccus that includes lineages capaple of causing infections such as meningitis, arthritis, endocarditis, septicemia, and pneumonia in pigs worldwide [1]. Disease-associated *S. suis* (DASS) can cause disease at any production phase, but infections are particularly common in young pigs (5–10 weeks old), with mortality rates reaching 2–5%, leading to significant economic losses for farmers [2]. A recent study reported an increase in *S. suis*-associated disease diagnoses at ISU-VDL [3], highlighting the growing burden of this pathogen within the US swine industry. In Europe, veterinarians report *S. suis* as one of the most impactful agents in swine production, costing between 0.60 and 1.30 EUR/ per pig [4–6]. Additionally, *S. suis* represents a key factor driving the use of antimicrobials on farms [4].

*S. suis* is highly diverse, with at least 29 serotypes and over 3050 sequence types (STs). Although multilocus sequence typing (MLST) and serotyping are common methods for distinguishing pathogenic from commensal strains, both have limitations [7, 8]. Serotyping alone is insufficient due to the pathogenic heterogeneity observed within serotypes. Furthermore, both serotyping and whole-genome sequencing (WGS) necessitate pure culture isolation which is labor-intensive and less sensitive. These constraints underscore the need for more efficient, culture-independent methods to detect DASS strains in healthy pigs prior to disease onset.

Commensal and DASS lineages often co-colonize the upper respiratory tract of pigs, but non-pathogenic strains typically dominate, outnumbering pathogenic ones by 100- to 1000-fold [9]. This imbalance, driven by accessory genes conferring metabolic flexibility, limits the utility of culture-based methods for studying DASS dynamics, as commensals may overgrow DASS strains in vitro. This competitive disadvantage makes the sole use of culture-based techniques unsuitable for investigating DASS dynamics. Piglets are typically colonized by *S. suis* shortly after or during birth, although those delivered via cesarean section may initially avoid colonization [10, 11]. While it is well-established that piglets are colonized by *S. suis* early in life, the prevalence and timing of colonization by DASS remain unclear. Effective control and prevention of DASS in swine farms require accurate detection of disease-causing strains within the herd, identification of carrier animals, along with discovery and elimination of reservoirs. To address these challenges, alternative approaches, such as those targeting genetic markers specific to DASS, are necessary to overcome the limitations of culture-based methods.

Recent advances in comparative genomics have led to the identification of a genomic island present in 99% of DASS strains and largely absent from non-DASS strains [12]. Leveraging this finding, a novel qPCR assay was developed targeting a gene within this island, *SSU_RS01130*, annotated in *S. suis* strain P1/7 (NCBI RefSeq: NC_012925.1), capable of reliably distinguishing most DASS from commensal strains. In this study, we employed the *SSU_RS01130*-based qPCR assay to investigate DASS transmission dynamics and to identify optimal sampling sites for DASS detection across two commercial swine herds. Specifically, we aimed to characterize the effect of parity, define effective sampling strategies, and evaluate DASS colonization dynamics in dams, piglets, and the environment during the lactation period.

## Materials and methods

### Overview

In this study, the transmission dynamics of DASS detection among dams and piglets during the lactation period was assessed by a prospective cohort study carried out in two sow farms. Two target genes in qPCR assays were used to analyze *S. suis* species colonization (*recN* gene) and DASS colonization (*SSU_RS01130* gene) in swine and environmental samples from dams and piglets at various time points post-farrowing.

### Quantitative PCR target genes and conditions

A qPCR assay was developed for culture-independent detection of DASS in swine samples was used to screen all study samples for the detection of DASS. The qPCR was based on the *SSU_RS01130* gene, recently associated with pathogenic lineages and absent in non-clinical or commensal lineages [12]. Thus, serving as a virulence-associated marker. The *recN* qPCR assay was developed at the Iowa State University Veterinary Diagnostic Laboratory (ISU-VDL). The development of the two qPCR assays followed the MIQUE guidelines [13].

The *recN* and *SSU_RS01130* qPCRs were run independently on all samples, i.e., in a singleplex format. The two assays were run in a 20 µL total reaction volume, containing 5 µL of TaqMan^®^ Fast Virus 1-Step Master Mix with the added AmpliTaq^®^ 360DNA Polymerase (5U/µL) (Thermo Fisher Scientific, Waltham, MA. USA), primers (0.4 µmol), probes (0.2 µmol), 8.135 µL of water, and 5µL of the DNA template (Table 1). Assays for both genes were run to 40 thermal cycles (Ct) on the automated Applied Biosystems^®^ 7500 Real-Time PCR (Thermo Fisher Scientific, Waltham, MA, USA). The amplification conditions were as follows: 50 °C for 5 min; 95 °C for 20 s, 40 cycles for 95 °C for 15 s followed by 60 °C for 60 s. Each run included a non-template control (PCR-grade H_2_O) and positive control (DNA from *S. suis* pure culture, confirmed to carry the target genes). Positive control strain was confirmed to be *S. suis* using matrix-assisted laser ionization-time of flight mass spectrometry (MALDI-TOF) and carried the *SSU_RS01130* and *recN* genes using whole-genome sequencing. The qPCR results were analyzed using the SDS1.5.2 software (Applied Biosystems, Carlsbad, CA, USA).

**Table 1:** Primers and probes for *SSU_RS01130* and *RecN* qPCR assays

Target Gene	Oligo Name	Sequence (5′→3′)	Amplicon Length
*SSU_RS01130*	SSU_RS01130_Fw	GAGAGATTGGTCATACAGTA	92 bp
	SSU_RS01130_Probe	VIC-TCCACATTCACAAGAAGGTCCGT-MGBNFQ	
	SSU_RS01130_Rev	CCAAGTATCAGAAATATAAGTTTG	
*recN*	recN_Fw	CTTTTGGACAGTTTCGGAGAAGA	107 bp
	recN_Probe	FAM-AAGACCGTTATCAGACAAC-QSY	
	recN_Rev	TTTTGCTTTTCAAGAACTCGTTTG	

### Analytical and diagnostic sensitivity and specificity

To determine the limit of detection of the *recN* and *SSU_RS01130* gene qPCR assays, a 150 bp GBlock containing *recN* or *SSU_RS01130* primers and probe binding sites were obtained (Integrated DNA Technologies, Coralville, IA, USA). A GBlock is a double-stranded synthetic DNA fragment. The synthetic DNA was then hydrated in Tris-EDTA buffer (Invitrogen™, Waltham, MA USA) to make a concentration of 25ng/ µL. The hydrated synthetic DNA was used to calculate the starting copy number/ µL. To generate a standard curve, ten-fold serial dilutions of the GBlock DNA for the assay containing 1 × 10^7^ copy number/ µL starting concentration for *recN* and 1 × 10^9^ copy number/ µL starting concentration for *SSU_RS01130* gene. Four independent qPCR runs, each with four replicates, were done on different days to determine the assay repeatability and reproducibility. The mean Ct, standard deviation, and coefficient of variation (CV) were calculated. The detection limit was obtained using the Ct of the lowest dilution, which gave consistently positive results. Two-fold dilutions were also tested across 25 repeats per dilution, with a Ct cut-off of < 37 determined as optimal for both genes, based on the 95% positivity threshold.

For the analytical specificity, the *recN* and *SSU_RS01130* gene primers and probe were in-silico tested for specificity using the standard settings search tool [14]. Futhermore, the following non-target species were tested for both *SSU_RS01130* and *recN* qPCRs; *Bacillus Cereus* 10876, *Actinobacillus suis* 33415, *Actinobacillus equuli* 19392, *Staphylococcus aureus* (ISU-VDL), *Salmonella choleraesuis* 6958, *Pasturella multocida* 12948, *E. coli 25,922*,* Mycoplasma hyosynoviae* (ISU-VDL), *Mycoplasma hyopneumoniae* (ISU-VDL), *Mycoplasma flocculare* (ISU-VDL), *Glaesserella parasuis* 19417, *Streptococcus porcinus* (ISU-VDL), and *Streptococcus porci* (ISU-VDL), *S. suis* serotype 22 (*n* = 3) and serotype 34 (*n* = 1) which are now independent species [15, 16]. All these strains were generously provided by the ISU-VDL.

To assess the diagnostic sensitivity of the assays, 208 whole-genome sequenced *S. suis* isolates were selected from a larger ISU VDL global collection (*n* = 1600). These isolates encompassed the most frequently detected serotypes and sequence types in the database, all with an average nucleotide identity (ANI) of at least 95% [17], and were tested for the presence of the *recN* gene. A subset of these *S. suis* isolates (*n* = 176), identified as part of pathogenic lineages based on MLST, core genome phylogeny, and the presence of virulence-associated markers, was tested for the presence of the *SSU_RS01130* gene (Additional File 1). Additionally a total of 60 tonsil swabs from expected DASS-positive pigs (nursery age pigs from *S. suis* affected flows) were tested with both assays.

For the diagnostic specificity evaluation, 30 tonsil swabs from cesarean-derived, colostrum-deprived (CDCD) pigs (http://www.premierbiosource.com/), and 60 tonsil swabs from specific pathogen-free (SPF) pigs (https://struvelabs.com), were tested for the presence of the *recN* and *SSU_RS01130* genes. Furthermore, the presence of the *SSU_RS01130* and *recN* genes was investigated through in-silico testing in a collection of *Streptococcus suis* isolates (*n* = 464) from clinical cases, which were whole-genome sequenced and submitted to the ISU-VDL. These isolates, representing diverse serotypes and sequence types (Additional File 2), were categorized as either pathogenic or commensal based on a recent publication [12].

### Study farms

The study farms were two 5600-head farrow-to-wean sow herds located in southern Wisconsin, managed by the same production company (Table 2). Each had a distinct endemic and clinical profile, based on historical diagnostic information and gilt genetic sourcing. While both farms were stocked from the same genetic company, they had been sourced from distinct genetic pyramids. Historical diagnostic data from each farm were compiled based on records of laboratory-confirmed *S. suis* cases. Mortality percentages were estimated by calculating the number of piglets that died with confirmed *S. suis* disease (based on necropsy findings, qPCR confirmation and whole-genome sequencing) divided by the total number of piglets in the batch. On Farm 1, recurrent cases caused by serotype 1 ST1 were estimated to account for 2–3% mortality, while on Farm 2, cases associated with serotype 2 ST28 accounted for < 1% mortality. All diagnoses were made by the attending veterinarian in conjunction with submissions to the Iowa State University Veterinary Diagnostic Laboratory (ISU-VDL) over the 12 months prior to study initiation. Both farms were porcine reproductive and respiratory syndrome (PRRS) and *M. hyopneumoniae*-naïve. Farm 1, provided ST1 *S. suis* vaccine to gilts at 22 and 26 weeks of age (Table 2). Prophylactic treatment was not allowed during the study, but therapeutic treatments were recorded when applied.

**Table 2:** Description of swine farms

	Farm 1	Farm 2
Herd size	5600	5600
Farm location (state)	Wisconsin	Wisconsin
Housing style	Crate gestation	Pen gestation
Production type	Continuous flow farrow-to-wean sow farm	Continuous flow farrow-to-wean sow farm
Gilt source	Source A	Source B
Replacement rate	55%	55%
PRRS status	Negative	Negative
M. hyopneumoniae status	Negative	Negative
Predominant *S. suis* type	*S. suis* serotype 1 ST1 positive	*S. suis* serotype 2 ST28 positive
Antibiotic treatment	No prophylactic antibiotic medication on sow or piglet	No prophylactic antibiotic medication on sow or piglet
*S. suis* Vaccine protocol	*S. suis* vaccine for gilts at 22 and 26 weeks of age	No *S. suis* vaccination pre-farrow or to the piglets
Liveborn (avg)	14.5	14.9
Weaning age (days)	21	21
Pre-weaning mortality rate (%)	12.5%	13.3%

### Study design and sample collection

Fifty dams per farm (25 parity zero and 25 P3+) from the same farrowing week were randomly selected and enrolled in the study. The average parity of enrolled sows was 6.2 on Farm 1 and 4.7 on Farm 2. For each dam, a tonsil scrape, nasal and vaginal swab sample was collected (HydraFlock, ITK diagnostics, Uithoon, Netherlands) at farrowing, and only tonsil swabs were collected at weaning. On farm 1, additional dam samples were collected at farrowing, including fecal swabs, farrowing crate swabs (environment), and udder swabs. On the same day of farrowing, up to 14 piglets were conveniently selected from each of the 50 litters on both farms. Piglets were double ear-tagged, and one tonsil swab was collected from each piglet within 12–24 h post-birth. The same piglets were sampled again at 7 and 21 days of age. Tonsil scraping was performed as previously described [19]. Briefly, the dams were physically restrained using a snare and a mouth speculum was used to open the oral cavity, and a long handled spoon was introduced into the oral cavity. A laryngoscope was used to guide the spoon and visualize the palatine tonsils. The spoon was utilized to scrape the tonsils and obtain an exudate. A sterile swab was then used to collect the exudate from the spoon. Nasal samples from the dams were collected by inserting a sterile swab deep into the nostril and rotating it for 5 s (https://www.securepork.org/training-materials/disease-monitoring-sample/nasal-swabs/). Rectal and vaginal swabs were collected by rotating a swab clockwise and counterclockwise at the respective sites. Environmental samples, which specifically refer to the surfaces of the dam’s farrowing crate, and udder samples were collected using Swiffer pads soaked in phosphate-buffered saline (PBS), with the pads wiped across either the farrowing crate surfaces or the udder. Samples were refrigerated upon collection and stored at 4 °C overnight until laboratory processing the following day.

### Sample processing and DNA extraction

A total of 1mL sterile PBS was added to each swab sample, vortexed vigorously for 10 s, aliquoted into two samples, labeled and frozen at -80 °C until DNA extraction. The DNA was extracted using the commercial kit MagMAXTM-96 Pathogen RNA/DNA kit (Applied Biosystems, Carlsbad, CA) on a Kingfisher-Flex instrument (Thermo fisher scientific, Waltham, MA USA) following the manufacturer’s instructions. Nucleic acid was eluted into a 90 µL elution buffer. The DNA was then stored at -80 °C until it was used for the qPCR testing.

### Statistical analysis

Data management, as well as descriptive and inferential analyses, were conducted using Microsoft Excel^®^ and R (version 4.0.0, R Core Team, 2020). A linear mixed-effects model was fitted using the lme4 package in R, including fixed effect the gene (categorical, with levels *recN* and *SSU_RS01130*), and random effects the sow and farm identifiers. The assumptions of linear models were verified using residuals vs. fitted values plot (which showed no systematic patterns), Q-Q plot (which confirmed normality of residuals), and Breusch-Pagan test to check for constant variance (which resulted in *p* = 0.10, indicating no evidence of heteroscedasticity).

The comparison of odds ratios and detection rates of DASS in sows between sample types, sampling stages, and group were done using logistic regression models separately for each of the two farms. Individual DASS positivity in piglets was compared between group and sampling days using mixed-effect logistic regression including sow identifier as random effects separately for each of the two farms. Litter-level prevalence was calculated as the proportion of piglets testing positive for DASS within each litter, i.e., the number of DASS-positive piglets divided by the total number of piglets sampled in that litter at each time point. Finally, to determine if dam positivity was a predictor for litter prevalence, a Poisson regression model separately by farm was used with dam status, group, and day (and interaction when significant) as explanatory. The statistical significance was interpreted when p- value was lower than 0.05 and Tukey-Kramer test was used for pairwise comparisons among groups.

## Results

### RecN and SSU_RS01130 qPCRs assays

#### Analytical sensitivity and specificity

The detection limit for both gene assays was determined to be 10 copies per µL of extracted DNA, corresponding to the highest dilution yielding at least 95% positive results. The standard curve for the *recN* gene produced a linear regression line with the equation y = -3.4703x + 43.208, with an R² value of 0.9987, and an assay efficiency of 94.16%. Similarly, the standard curve for the *SSU_RS01130* gene generated a linear regression line with the equation y = -3.35855x + 42.182, with an R² value of 0.9974, and an assay efficiency of 90.06%. Both assays demonstrated inter-assay and intra-assay variability with a coefficient of variation less than 10%.

The in-silico analysis of the *recN* and *SSU_RS01130* gene primers and probes using BLAST did not identify any similar GenBank sequences outside of the *S. suis* species. All non-target species or strains, including serotype 22 and 34 isolates (reclassified as distinct species), tested negative for both genes.

#### Diagnostic sensitivity and specificity

A subset of 208 *S. suis* isolates from the ISU VDL global collection (*n* = 1600), representing the most frequently detected serotypes and sequence types, was tested for the presence of the *recN* gene. Of these, 163 isolates, previously identified as pathogenic based on both recent findings and well-established pathogenicity criteria, also tested positive for the *SSU_RS01130* gene by qPCR [12]. Among the 208 isolates, 45 isolates from commensal/opportunistic lineages, were negative for *SSU_RS01130* (Additional File 1) [12].

In-silico analysis of 464 whole-genome sequenced isolates (*recN* positive) confirmed that 387 strains belonging to pathogenic lineages carried the *SSU_RS01130* gene, while 77 isolates corresponding to commensal lineages tested negative (Additional File 2). Additionally, all 60 tonsil swabs from post-weaning commercially reared pigs were positive for the *SSU_RS01130* gene.

Tonsil swabs from 60 specific pathogen-free (SPF) pigs tested negative for both *SSU_RS01130* and *recN* genes by qPCR. All 30 tonsil swabs from cesarean-derived, colostrum-deprived (CDCD) pigs tested positive for *recN* but negative for *SSU_RS01130* by qPCR.

### Detection of S. suis based on the recN real-time qPCR

All dam tonsil and nasal samples tested positive for the *recN* gene across all sampling days on both farms, with the exception of one sow from Farm 1 that was negative in the tonsil swab at farrowing. The average Ct value was 31.07 (range, 27.4-37.59) and 31.28 (range, 28.14–36.59) on Farm 1 and Farm 2, respectively. Additionally, all 50 udder, fecal, and environmental samples collected from Farm 1 were *recN* positive. For both farms, 90% (45/50) of vaginal swabs tested *recN* positive. All tonsil samples from piglets except two on farm 2 were *recN* positive (*n* = 3649).

### DASS detection via SSU_RS01130 qPCR

#### Dam-level detection by parity and sample type

**Fig. 1:**
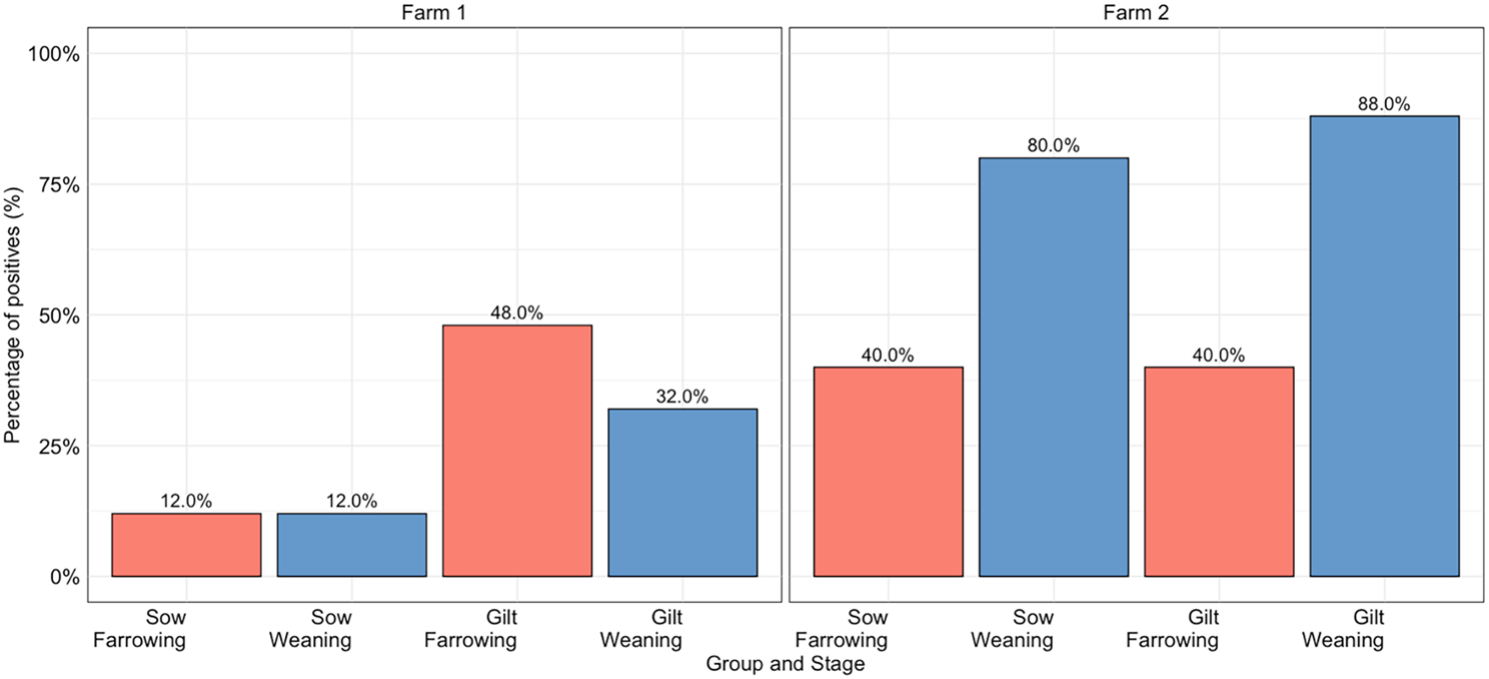
Detection of DASS in dam tonsil samples on day 1 (farrowing) and day 21 (weaning)

For Farm 1, DASS detection in tonsil samples at farrowing was 30% (15/50) and numerically decreased to 22% (11/50) by weaning (Day 21) (*p* > 0.05). In contrast, dam DASS detection in Farm 2 was 40% (20/50) at farrowing, statistically increasing to 84% (42/50) by weaning (*p* < 0.05) (Fig. 1).

In Farm 1, 36.0% (9/25) of gilts and 80.0% (20/25) of sows remained negative throughout based on tonsil samples alone. In comparison, 16.0% (4/25) of gilts and 4.0% (1/25) of sows were consistently positive for DASS throughout the study period. In Farm 2, 4.0% (1/25) of gilts and 12.0% (3/25) of sows remained negative in the tonsil throughout, while the majority were either positive at weaning (Day 21) only, or continously positive (Fig. 1; Additional File 3).

The odds of DASS detection in tonsils was 82% lower in sows compared to gilts on Farm 1 (*p* < 0.05). However, on Farm 2, the detection of DASS in tonsils gilts and sows was not significantly different (Fig. 1).

In tonsil swabs, the estimated Ct value for the *SSU_RS01130* gene was 3.37 (standard error 0.215) units higher than for the *recN* gene (*p* < 0.001; Additional file 4). On Farm 1, DASS-positive dams had higher *SSU_RS01130* Ct values than those on Farm 2, particularly at farrowing. However, no significant differences in *SSU_RS01130* Ct values were observed between DASS-positive gilts and sows (*p* > 0.05, Additional File 4).

**Fig. 2:**
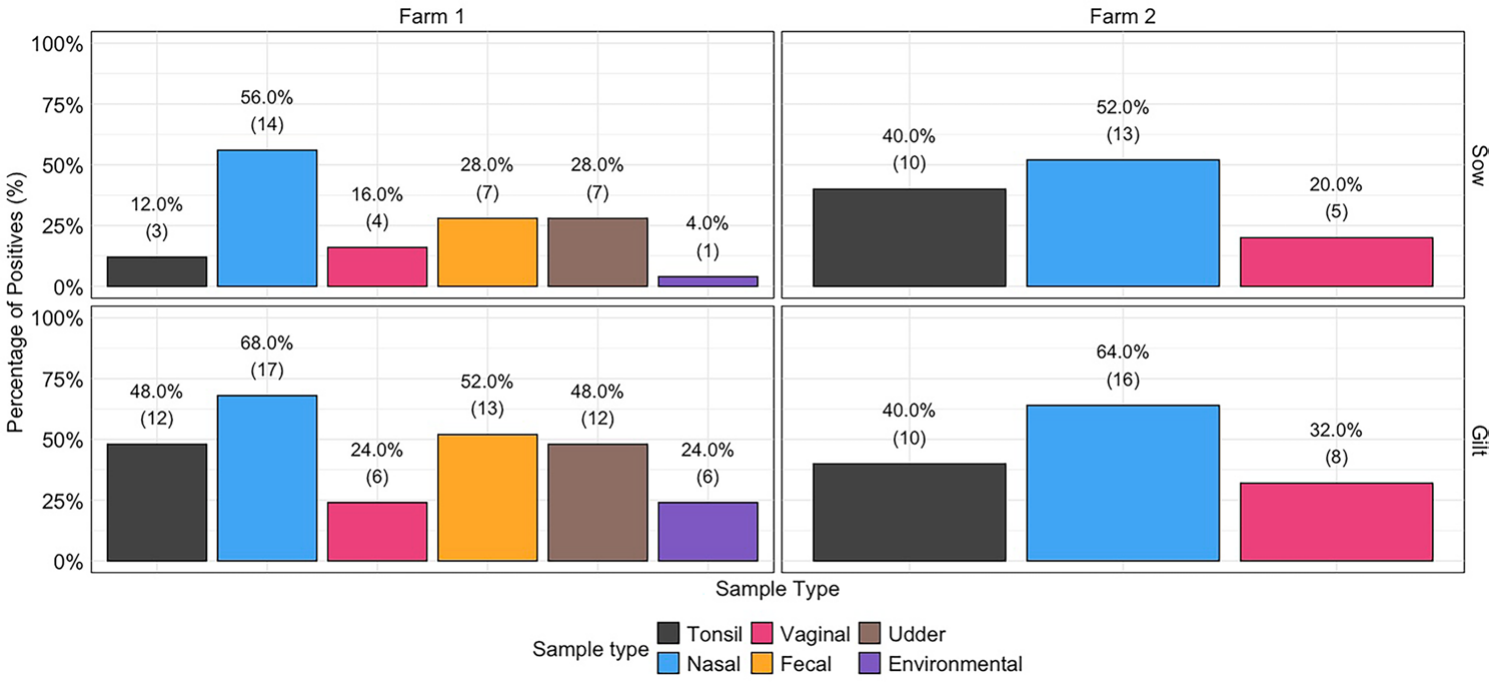
Percentage of positive samples by type, farm, and sow parity group

DASS detection varied by sample type, parity, and farm (Fig 2). Farm 2 had higher overall detection rates compared to Farm 1. Across both farms, fecal and nasal samples showed the highest positivity, while environmental and vaginal samples had lower detection rates. In Farm 1, gilts were associated with 2.58 (95% CI 1.62–4.14, *p* < 0.001) times higher odds of being DASS positive compared to sow. In contrast, no difference was detected between gilts and sows in Farm 2 (*p* > 0.05).

In Farm 1 gilts and sows, DASS detection odds did not significantly differ between tonsil, fecal, udder, or vaginal samples (*p* > 0.05). Nasal samples had the highest detection odds, showing a 4.15 (95% CI 1.79–10.05, *p* < 0.05) times higher compared to tonsil samples. While environmental samples were associated with 64% lower odds (odds ratios 0.36, 95% CI 0.12–0.99, *p* = 0.05) than tonsil samples. Fecal and nasal samples also had significantly higher detection odds than environmental samples (*p* < 0.05). No differences were observed between environmental and vaginal samples, fecal and nasal samples, or fecal and vaginal samples. Notably, nasal samples had 13 times higher detection odds compared to vaginal samples (*p* > 0.05).

In Farm 2, there was a trend for the association of DASS detection odds between tonsil and nasal samples. That is, the odds of DASS detection in nasal samples was 2.08 times higher (95% CI 0.94–4.70) than tonsil samples (*p* = 0.07). Otherwise, there was no statistical difference between tonsil and vaginal samples (*p* > 0.05).

### Piglet and litter-level detection patterns

A total of 629 piglets from Farm 1 and 631 from Farm 2 were sampled 12–24 h after birth. Pre-weaning mortality rates for the study pigs was 6.9% and 6.0%, respectively. Both farms showed a similar temporal pattern in DASS detection: a decrease on Day 7 relative to Day 1, followed by an increase on Day 21. Despite this shared trend, overall DASS detection was consistently lower on Farm 1 compared to Farm 2 across all time points. Notably, only gilt piglets on Farm 2 did not exhibit higher DASS detection on Day 21 relative to Day 1, differing from the pattern observed in other groups (Fig. 2). Ct values for the species-specific *recN* gene remained low across farms and time points (mean 25.3–28.5), confirming widespread *S. suis* colonization in piglets. In contrast, *SSU_RS01130* Ct values were higher (mean 30.6–34.6), indicating lower abundance of disease-associated *S. suis* (DASS). On Farm 2, particularly in gilt piglets, *SSU_RS01130* Ct values decreased over time, suggesting increasing DASS colonization during lactation. This trend was less pronounced on Farm 1, where overall DASS burden was lower. These findings highlight higher DASS prevalence in gilts, especially on Farm 2 (Additional File 5).

**Fig. 3:**
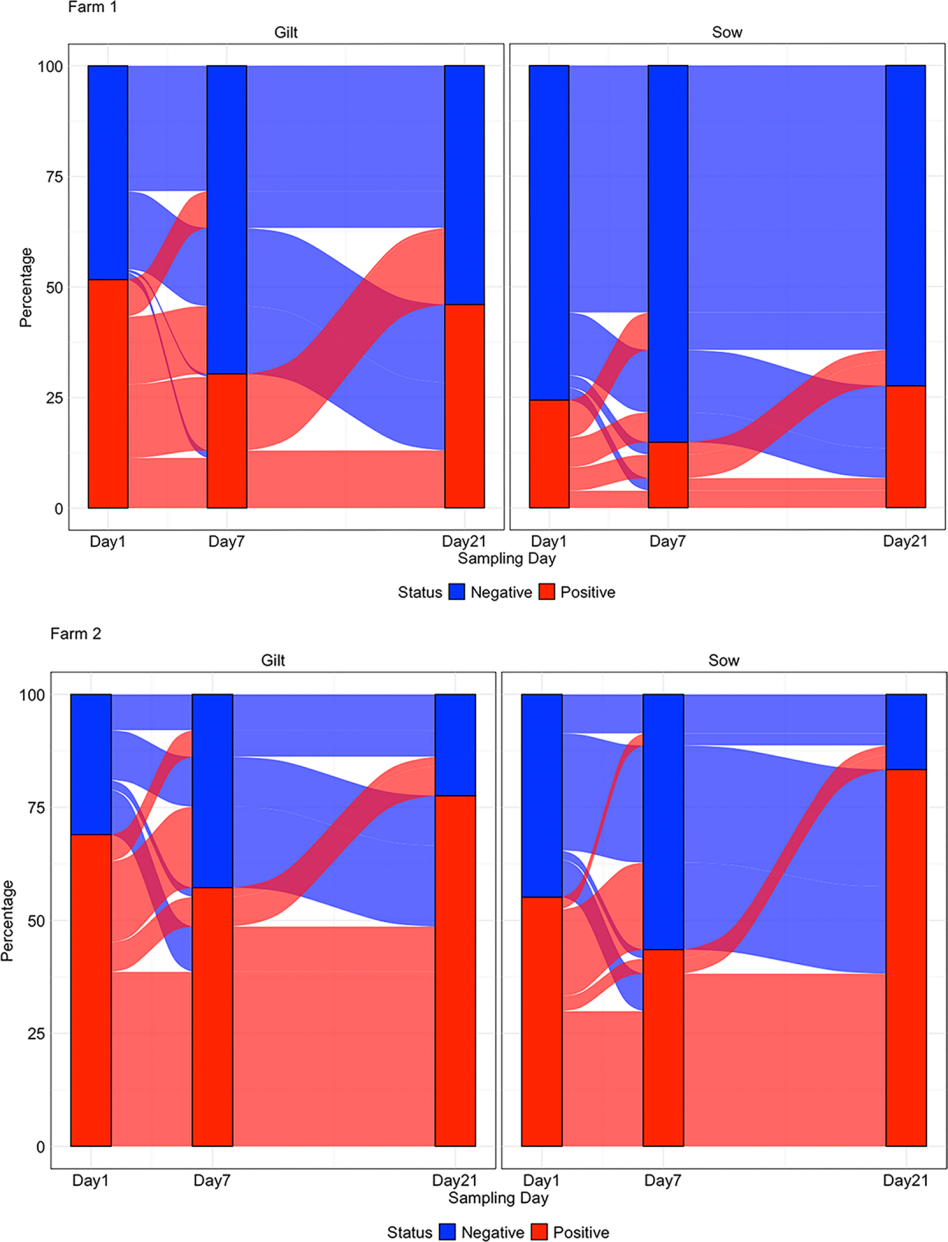
DASS detection in piglet tonsil swabs by sampling day and parity on Farm 1 and Farm 2. Bar heights represent the average proportion of positive and negative piglets at each time point (Days 1, 7, and 21). Ribbons indicate individual pig-level detection patterns across time

For farm 1, sow piglets had significantly lower DASS detection compared to gilt piglets, with estimated DASS detection rates of 34.6% (95% CI 19.4–53.2%) for gilts vs. 10.5% (95% CI 5-21.1%) sows in Farm 1 (*p* < 0.05) (Fig. 3). Although not significant, the estimated DASS detection rate was 76.0% for gilts (95% CI 62.6–85.7%) vs. 67.1% for sows (95% CI 52.1–79.3%) (*p* > 0.05) on farm 2. On Farm 1, 30.2% of gilt piglets and 57.2% of sow piglets remained negative throughout the study (Additional File 6). In contrast, 10.3% of gilt piglets and 3.7% of sow piglets consistently tested positive across all three sampling points. A total of 42% of piglets from gilts and 23.1% of piglets from sows showed fluctuating detection of DASS (Fig. 3). On Farm 2, between 8 and 12% of piglets remained negative, whereas 37% of gilt piglets and 30% of sow piglets consistently tested positive for the DASS gene. A total of 35.9% of piglets from gilts and 27.9% of piglets from sows showed fluctuating DASS detection.

At the litter-level, Farm 1 had an overall DASS prevalence of 32.7%, while Farm 2 exhibited a higher prevalence of 64.97%. On Farm 1, 10 litters remained consistently negative throughout the study (Additional File 6). Among gilt litters, most exhibited either decreasing or fluctuating patterns of DASS detection over time, with only 1 litter remaining consistently high (> 70%) or low (< 30%). Sow litters in Farm 1 predominantly displayed consistently low detection rates, with occasional increases or decreases observed. In Farm 2, litters displayed a higher prevalence of consistently high and fluctuating detection patterns compared to Farm (1) Notably, several litters had a sustained high detection rate (e.g., 100% across all time points) in Farm (2) In sow litters, the most common patterns were increasing and consistently high, with a few showing decreasing or fluctuating trends.

The association between dam DASS positivity either by individual sample type and DASS positivity in piglets was evaluated. On Farm 1, dam positivity in fecal, environmental, vaginal, and udder samples was significantly associated with piglet DASS positivity (*p* < 0.05). In the final multivariate model (e.g., all samples types included together, through a backward process, and sample types were kept wih *p* < 0.05), dams with positive DASS udder samples were 13.68 (95% CI 5.99–31.26) times more likely to have piglets with DASS detection at birth compared to dams with negative udder samples (*p* = 0.0001). In contrast, on Farm 2, nasal and tonsil DASS positivity in dams showed no significant association with piglet tonsil DASS positivity (*p* > 0.05). Regarding the association dam DASS positivity in at least one sample type and DASS positivity in piglets in Farm 1, the interaction between dam parity and positivity in at least one sample type significantly increased the odds of DASS piglet positivity. Specifically, gilts that tested positive in at least one sample type had 53.42 (95% 3.25–87.52) times higher odds of having DASS-positive piglets at Day1 (*p* = 0.005).

**Fig. 4:**
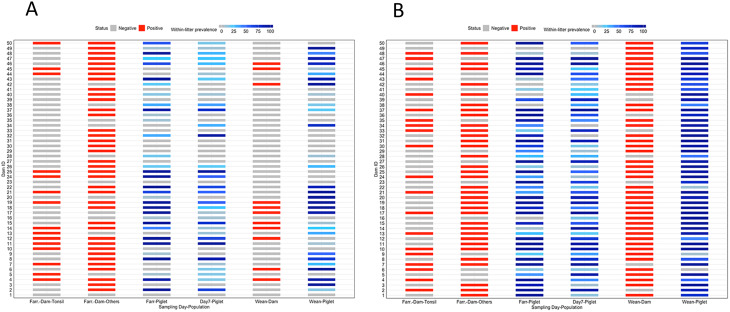
Association between dam DASS colonization and litter prevalence at farrowing, day 7, and weaning (day 21) for Farm 1 (A) and Farm 2 (B). Columns 1 and 5 in each graph represent DASS detection in the tonsils of dams at farrowing and weaning, respectively. Column 2 reflects DASS detection in at least one dam sample type other than the tonsils. Columns 3, 4 and 6 indicate litter DASS prevalence at farrowing, day 7, and weaning, respectively. Dams 1-25 correspond to gilts, while dams 26-50 are sows for each farm

At the litter level, dam positivity in tonsil was a predictor of litter prevalence (Fig. 4). Litters from positive gilts had a 37% higher (95% CI 9.8%, 61%) prevalence than those from positive sows (*p* < 0.05, Poisson regression) in Farm 1. On Farm 2, however, dam positivity across all tested sample types (tonsil, vaginal, and nasal) was not significantly associated with piglet DASS positivity (*p* > 0.05), and dam positivity did not predict litter prevalence on Farm 2 (*p* > 0.05).

## Discussion

Effective control and prevention of *S. suis*-associated infections in swine herds rely on accurately identifying disease-causing strains or carriers within the population [1]. Detecting carriage of Disease-Associated *S. suis* (DASS) in clinically healthy animals remains challenging due to the limitations of existing assays and the difficulty of isolating DASS strains from the complex tonsillar microbiota. A molecular tool capable of detecting a gene exclusive to pathogenic strains would significantly enhance surveillance and control efforts in live animals.

Identifying *Streptococcus* species remains challenging due to extensive phenotypic and genetic diversity, and misclassification is commonly encountered within diagnostic laboratories [19]. Although MALDI-TOF MS provides a rapid and cost-effective solution for identification [20, 21], it requires pure cultures and can occasionally yield inaccurate identifications [22]. Molecular assays targeting the *recN* gene have emerged as preferred methods for species-level confirmation over traditional 16 S rRNA or *gdh* PCR assays [23]. To overcome these limitations, novel molecular assays targeting specific genetic markers are necessary for improved pathogen surveillance. Recently, the *SSU_RS01130* gene, a marker associated with pathogenic *Streptococcus suis* lineages, demonstrated consistent reliability across diverse geographic regions [12]. In this study, we validated two novel qPCR assays for use in both pure cultures and live swine samples: the first targets the *recN* gene for accurate species-level detection, while the second specifically targets *SSU_RS01130* for identifying DASS. Both assays exhibited high diagnostic accuracy and efficiencies within recommended standards [13]. The species-specific *recN* assay served as a control for sampling adequacy, while the *SSU_RS01130* assay identified samples carrying the gene marker enriched among DASS lineages, enabling herd-level surveillance and potential source tracking. Furthermore, the *SSU_RS01130* assay offers a rapid molecular proxy for differentiating strains with increased disease-associated potential in pure cultures, thereby supporting both clinical diagnostics and control program implementation.

The longitudinal study of DASS during lactation revealed distinct infection patterns across farms. On Farm 2, DASS prevalence in sows increased substantially from farrowing to weaning—a trend not observed on Farm 1. Similarly, piglets on Farm 2 exhibited consistently higher DASS prevalence at all sampling points compared to those on Farm 1. While this study was not designed to directly compare farms, these differences suggest that management practices, parity distribution, or DASS strain profiles may influence colonization dynamics and warrant further investigation. For instance, strain-specific differences in virulence and transmission could explain both the historical clinical impact and the observed prevalence patterns. Farm 1, despite experiencing higher *S. suis*-associated mortality, had lower DASS prevalence, suggesting that virulence may not necessarily correlate with colonization levels. This counterintuitive finding may indicate that highly virulent strains like ST1, despite their capacity to cause disease, might colonize less efficiently than strains like ST28. Transmission efficiency, host immune responses, or microbial competition could underlie this dynamic and deserve further exploration. Supporting this, piglet innate immune responses have been shown to vary by strain virulence [24]. Additionally, prior vaccination of gilts on Farm 1 may have influenced the dynamics of *S. suis* colonization.

Gilts consistently showed higher positivity rates, potentially linked to their less mature immune systems and lower specific immunoglobulin levels. This observation underscores the importance of targeted gilt management strategies to reduce pathogen transmission. Shakhov et al. (2021) [25] reported a postpartum decline in serum immunoglobulin levels, which could increase susceptibility to colonization. Additionally, environmental contamination may contribute to re-colonization during lactation, given DASS’s persistence in the environment [26, 27]. Interestingly, on Farm 1, gilts were more likely to be DASS-positive in tonsil scrapes compared to sows. This aligns with existing literature suggesting that gilts are more susceptible to pathogens due to their less mature immune system and limited lifetime immune experience [28]. On average, sows in Farm 1 were older (parity 6.2) than those in Farm 2 (parity 4.7), which may have contributed to a greater contrast in DASS colonization between gilts and sows on Farm 1. Notably, this parity-related difference was observed only on Farm 1, underscoring the need for cautious interpretation. Farm-specific factors such as environmental conditions, management practices, or endemic pathogen pressure may have influenced these findings and warrant further investigation [29]. One relevant factor in Farm 1 was the use of a *Streptococcus suis* vaccine targeting ST1, administered to gilts during the growing phase. Although no pre-farrow booster was given and immunity likely waned by farrowing, prior vaccination may still have influenced colonization dynamics. A recent study reported that sow vaccination reduced *S. suis* colonization in piglet tonsils up to three weeks post-weaning, suggesting immune exclusion could modulate colonization risk [30]. However, other studies have found no significant differences between vaccinated and unvaccinated sows [31], highlighting the complexity of maternal immunity in *S. suis* control. The lower DASS prevalence observed in Farm 1 may partially reflect residual effects of gilt vaccination, though the magnitude of this impact remains uncertain and warrants further investigation.

Additionally, sow housing differed between farms: Farm 1 utilized crated gestation, while Farm 2 employed group (pen) gestation. Group housing enables more direct contact among sows, which may influence *Streptococcus suis* transmission dynamics and microbial exposure prior to farrowing [32]. However, the impact of increased sow-to-sow transmission or colonization on downstream disease risk remains unclear. Further research is needed to understand how these housing-related differences affect DASS colonization patterns and their relevance to disease outcomes.

While overall DASS detection rates were similar between nasal and tonsil samples on Farm 2, nasal samples showed significantly higher positivity on Farm 1.This suggests that nasal sampling may be more sensitive in certain settings. However, in both farms, a proportion of DASS-positive dams were identified only through tonsil sampling at Day 1 (farrowing): 20% (3/15) on Farm 1 and 38% (8/21) on Farm 2 tested negative by nasal swabs despite being tonsil-positive. These findings indicate that reliance solely on nasal samples could lead to under-detection of DASS carriers in some cases. Although nasal swabbing is less invasive and more practical than tonsil sampling, these results highlight the need for further validation to determine the optimal sample type for accurate DASS surveillance in live animals.

In dams, both tonsil and nasal samples were more sensitive in detecting DASS compared to vaginal swabs. However, the detection of DASS-positive vaginal samples indicates a potential route for piglet colonization during the birthing process. This aligns with previous reports of pathogenic *S. suis* strains in vaginal samples [33]. In this study on farm 1, dam positivity in vaginal samples was significantly associated with piglet positivity, whereas tonsil and nasal samples showed no such association. This finding suggests that vertical transmission during parturition may be a critical pathway for early DASS colonization in piglets. This study also detected DASS in additional sample types, including udder wipes, farrowing crate wipes (environment), and fecal swabs. These results suggest that such sources may serve as potential reservoirs for piglet exposure and warrant further investigation.

Piglet colonization showed a clear parity effect on both farms, with piglets from gilts more frequently DASS-positive than those from sows. This likely reflects the lower immunoglobulin levels and quality of colostrum, a consequence of their lesser exposure to *S. suis* [34], which may leave piglets more vulnerable to early colonization. These findings, combined with the higher DASS detection in gilts on Farm 1, support the need for gilt-specific interventions, such as targeted vaccination or immune-priming strategies, to enhance colostrum quality and reduce vertical transmission. Notably, a recent study also found that lower-parity sows were more likely to have piglets affected by *S. suis*, reinforcing the importance of maternal immunity [29]. While some studies report a role for maternal antibodies in shaping piglet colonization and microbiota composition [30], others found no significant effects [31], highlighting the complex and context-dependent nature of maternal immune protection.

Despite the protective effects of colostrum, piglets on both farms were colonized with *S. suis* within 24 h of birth, as evidenced by detection of both *recN* and *SSU_RS01130* genes in tonsil samples. This finding is consistent with previous studies demonstrating rapid colonization after birth [11, 33, 35]. While early colonization is typically considered a risk factor for systemic disease, it may also play a dual role. Exposure to circulating DASS strains during the window of maternal antibody protection could help prime the piglets’ immune system to recognize and respond to these pathogens. This concept aligns with studies showing that early microbial exposure can enhance adaptive immune responses, particularly when balanced by passive immunity [36]. While early colonization may contribute to immune priming, the detection of DASS in vaginal swabs supports vertical transmission as a significant route of acquisition. This raises critical questions about balancing the potential protective effects of early exposure with the risks of systemic disease. Notably, lower DASS detection in Farm 1 during this early window suggests that piglets there may remain susceptible once maternal immunity wanes, potentially delaying the development of effective active immunity. Although sampling ended at 21 days of age, colonization is likely to increase post-weaning as maternal antibodies decline and piglets are exposed to DASS-positive peers in nursery environments [1].

These dynamics have important implications for DASS control. The higher colonization rates observed in gilts and their piglets highlight this group might be a key driver of early transmission within herds. Pre-farrowing interventions, such as improving colostrum quality and intake, implementing gilt-specific vaccination programs, using targeted antimicrobials, or applying parity segregation strategies, could help reduce the initial pathogen load passed to piglets. By limiting contact between high-risk gilt litters and lower-risk sow litters, parity segregation may reduce cross-litter transmission and dampen overall pathogen pressure within the farrowing environment.

In addition to gilt-targeted interventions, batch farrowing may offer a practical management strategy to reduce *S. suis* transmission. By aligning farrowing dates into distinct groups, farms can more effectively implement all-in/all-out practices, limit contact between age groups, and reduce continuous pathogen circulation. This approach could also facilitate targeted disinfection and downtime between farrowing groups, potentially minimizing environmental contamination, particularly relevant given our detection of DASS in crate surfaces, udders, and feces. Moreover, batch farrowing enables focused vaccination or antimicrobial strategies timed to specific production flows. Based on our data, where colonization persistence was evident throughout the lactation period, batch farrowing could help disrupt transmission cycles and may enhance the effectiveness of broader DASS control programs.

Medicated early weaning (MEW) as an elimination strategy for disease-associated *Streptococcus suis* (DASS) presents both opportunities and challenges [37]. By separating piglets from dams early and providing antimicrobial treatments, MEW aims to disrupt the transmission of DASS from maternal and potential environmental reservoirs. Findings from this study highlight several limitations of MEW for DASS elimination. The environment’s potential to serve as a reservoir for *S. suis*, suggests that complete elimination through MEW alone may be difficult. From an antimicrobial stewardship standpoint, it is important to underscore that MEW is not intended for routine disease control but rather as a short-term, targeted intervention within structured elimination programs. In farms with lower baseline prevalence like Farm 1, modifications to MEW could enhance effectiveness. Delaying weaning to Day 7 allows piglets to receive residual milk immunoglobulins and provides practical advantages, as piglets at this age are more robust and resilient. Prioritizing older-parity sows for MEW could further reduce piglet susceptibility due to potentially lower pathogen shedding. Finally, deep cleaning and disinfection of farrowing and nursery areas remain essential to minimize environmental reservoirs and reduce re-colonization risk. Future work should evaluate the feasibility and long-term impact of MEW-based strategies under diverse farm conditions, with particular attention to judicious antimicrobial use.

### Limitations

While the SSU_RS01130 gene serves as a marker associated with pathogenic *S. suis* lineages, it is not strain-specific and does not directly indicate the presence of a specific pathogen or predict clinical disease outcomes. Therefore, the observed detection patterns simply reflect the presence of a gene enriched among strains with pathogenic potential, rather than tracking the dynamics of a particular virulent clone. Moreover, the functional role of *SSU_RS01130* in virulence is not fully understood—some pathogenic strains may lack the gene, while some carriers may not cause disease. Despite these limitations, we found a strong association between *SSU_RS01130* and globally distributed, clinically relevant DASS lineages [12], supporting its utility as a molecular proxy for surveillance. The two PCR assays used in this study do not distinguish between live and dead bacterial cells; therefore, some environmental detections may have resulted from non-viable organisms. Finally, as this study was conducted on only two farms with differing management practices, extrapolation of the findings and conclusions to other farms should be approached with caution.

## Conclusion

Nearly all piglets were colonized with *S. suis* within 24 h of birth, though DASS prevalence was low and fluctuated over time. These findings highlight the importance of maternal immunity, hygiene, and colostrum management, particularly among gilts, in mitigating DASS colonization. No significant difference was observed in DASS detection between tonsils and nasal samples in dams on farm 2, suggesting that these two niches could be equally suitable for DASS surveillance in healthy pigs. The detection of DASS in other dam-related sample types and the dam environment indicates potential sources of DASS colonization in piglets, implying that improving hygiene may enhance overall prevention. While these results are specific to the two farms studied, they offer new insight into DASS transmission under field conditions. Importantly, the validated qPCR assay may serve as a culture-independent tool for monitoring DASS presence in future research and elimination programs. Further studies are needed to assess DASS persistence post-weaning and to evaluate interventions such as gilt acclimation, immunological strategies, and microbiota-based approaches.

## Supplementary Information

Below is the link to the electronic supplementary material.

Additional File 1

Additional File 2

Additional File 3

Additional File 4

Additional File 5

Additional File 6

## Data Availability

Raw data will be provided upon request from the corresponding author.

## Abbreviations

DASS: Disease-associated Streptococcus suis
MEW: Medicated early weaning
ISU-VDL: IOWA State University Veterinary Diagnostic Laboratory
MLST: Multilocus sequence typing
WGS: Whole-genome sequencing
ANI: Average nucleotide identity
PRRS: Porcine reproductive and respiratory syndrome
