# Supplementary material for: Disease-associated Streptococcus suis (DASS) in lactation: detection patterns and implications for control
Source: Porcine Health Manag. 2025 Nov 7;11:57. doi: 10.1186/s40813-025-00469-y (PMC12595806; doi:10.1186/s40813-025-00469-y)
Supplement: Supplementary file 3 — Additional File 3 [file 40813_2025_469_MOESM3_ESM.docx]

Dynamics of DASS tonsil carriage in dams by farm.

| **Farm** | **DASS status (Day)** | **Gilts % (n)** | **Sows % (n)** |
| --- | --- | --- | --- |
| **1** | Neg (1) - Neg (21) | 36.0% (9) | 80.0% (20) |
|  | Neg (1) - Pos (21) | 16.0% (4) | 8.0% (2) |
|  | Pos (1) - Neg (21) | 32.0% (8) | 8.0% (2) |
|  | Pos (1) - Pos (21) | 16.0% (4) | 4.0% (1) |
| **2** | Neg (1) - Neg (21) | 4.0% (1) | 12.0% (3) |
|  | Neg (1) - Pos (21) | 56.0% (14) | 48.0% (12) |
|  | Pos (1) - Neg (21) | 8.0% (2) | 8.0% (2) |
|  | Pos (1) - Pos (21) | 32.0% (8) | 32.0% (8) |

Neg = Negative, Pos = Positive
