# Supplementary material for: Disease-associated Streptococcus suis (DASS) in lactation: detection patterns and implications for control
Source: Porcine Health Manag. 2025 Nov 7;11:57. doi: 10.1186/s40813-025-00469-y (PMC12595806; doi:10.1186/s40813-025-00469-y)
Supplement: Supplementary file 5 — Additional File 5 [file 40813_2025_469_MOESM5_ESM.docx]

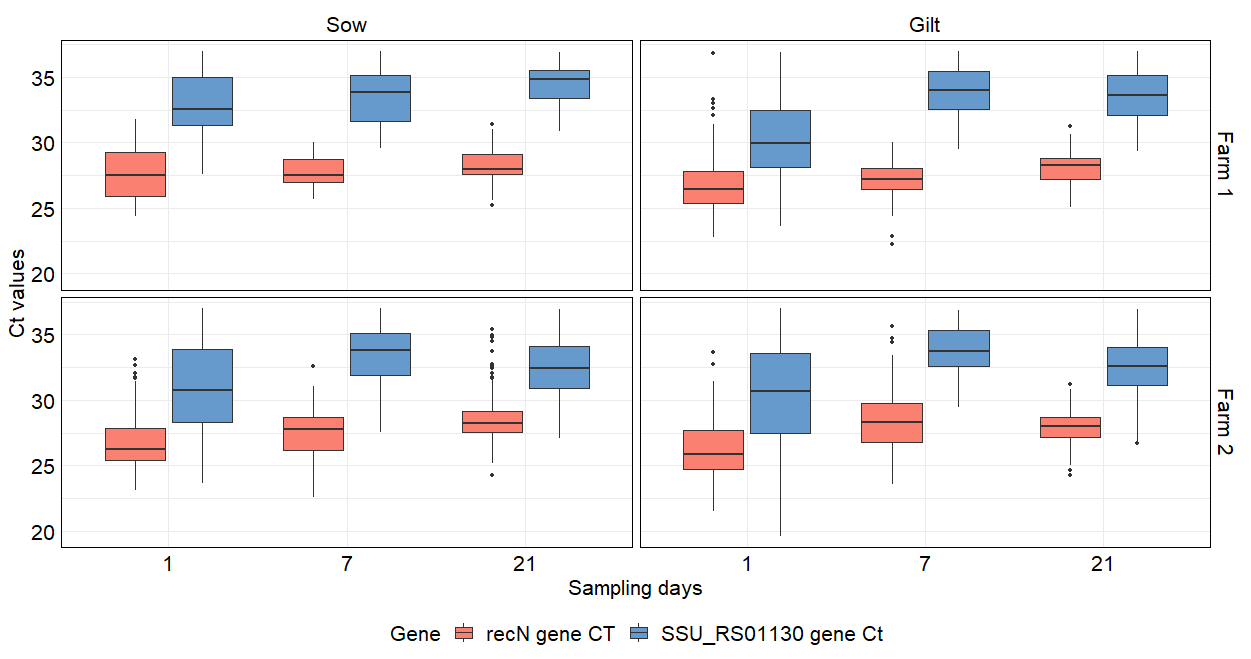


Ct values for *recN* and SSU_RS01130 gene-positive tonsil samples from piglets, grouped by farm and parity, at days 1, 7, and 21
