# Supplementary material for: Disease-associated Streptococcus suis (DASS) in lactation: detection patterns and implications for control
Source: Porcine Health Manag. 2025 Nov 7;11:57. doi: 10.1186/s40813-025-00469-y (PMC12595806; doi:10.1186/s40813-025-00469-y)
Supplement: Supplementary file 6 — Additional File 6 [file 40813_2025_469_MOESM6_ESM.docx]

Dynamics of DASS colonization in piglets by farm

| **Farm** | **Pattern** | **Gilts** | **Sows** |
| --- | --- | --- | --- |
| **Farm 1** | Neg (1) - Neg (7) - Neg (21) | 30.2% (100) | 57.2% (171) |
|  | Neg (1) - Neg (7) - Pos (21) | 16.0% (53) | 13.4% (40) |
|  | Neg (1) - Pos (7) - Neg (21) | 0.9% (3) | 2.7% (8) |
|  | Neg (1) - Pos (7) - Pos (21) | 1.5% (5) | 2.7% (8) |
|  | Pos (1) - Neg (7) - Neg (21) | 10.9% (36) | 9.0% (27) |
|  | Pos (1) - Neg (7) - Pos (21) | 13.9% (46) | 6.4% (19) |
|  | Pos (1) - Pos (7) - Neg (21) | 16.3% (54) | 5.0% (15) |
|  | Pos (1) - Pos (7) - Pos (21) | 10.3% (34) | 3.7% (11) |
| **Farm 2** | Neg (1) - Neg (7) - Neg (21) | 8.1% (25) | 11.8% (38) |
|  | Neg (1) - Neg (7) - Pos (21) | 10.4% (32) | 24.8% (80) |
|  | Neg (1) - Pos (7) - Neg (21) | 1.9% (6) | 1.9% (6) |
|  | Neg (1) - Pos (7) - Pos (21) | 9.4% (29) | 7.7% (25) |
|  | Pos (1) - Neg (7) - Neg (21) | 10.4% (32) | 4.6% (15) |
|  | Pos (1) - Neg (7) - Pos (21) | 16.8% (52) | 18.0% (58) |
|  | Pos (1) - Pos (7) - Neg (21) | 6.8% (21) | 3.4% (11) |
|  | Pos (1) - Pos (7) - Pos (21) | 36.2% (112) | 27.9% (90) |
